# Availability of Information About Lifestyle for Cancer Survivors in England: A Review of Statutory and Charitable Sector Organizations and Cancer Centers

**DOI:** 10.2196/cancer.3521

**Published:** 2015-03-09

**Authors:** Kate Williams, Abigail Fisher, Rebecca J Beeken, Jane Wardle

**Affiliations:** ^1^ Health Behaviour Research Centre Department of Epidemiology and Public Health University College London London United Kingdom

**Keywords:** cancer, survivorship, guideline, health behavior, lifestyle, diet, physical activity, body weight, smoking, alcohol drinking

## Abstract

**Background:**

Health behavior change following a cancer diagnosis has the potential to improve long-term outcomes. However, many patients do not receive professional advice about lifestyle and are therefore increasingly using the Internet to seek further information. The statutory and charitable sectors and cancer centers all play an important role in the provision of information and have been found to be favored by cancer survivors searching for information. However, to date there has been no systematic evaluation of the lifestyle information available online for cancer survivors.

**Objective:**

The purpose of this review was to identify the lifestyle information provided for cancer survivors by statutory and charitable sector organizations and cancer centers in the United Kingdom. We aimed to identify information on tobacco, physical activity, diet, weight, and alcohol designed for people who have been diagnosed with breast, prostate, or colorectal cancer.

**Methods:**

The National Health Service (NHS) website was the focus of the search for information provided by the statutory sector. Cancer centers were identified from the Organization of European Cancer Institutes and an Internet search, and charitable sector organizations were identified by searching the Charity Commission database. The three largest generic, breast, prostate, and colorectal cancer charitable organizations were included. A systematic search of the organizations was conducted to identify lifestyle information for cancer survivors.

**Results:**

Ten organizations had some lifestyle information for cancer survivors on their websites. The Christie NHS Foundation Trust, Macmillan Cancer Support, and Prostate Cancer UK had the most comprehensive guides, covering physical activity, diet, weight management, smoking, and alcohol. The NHS website did not provide any information but had a link to Cancer Research UK’s information about diet. Eight organizations suggested talking to a health professional before making any changes.

**Conclusions:**

The majority of organizations included in this review would benefit from updating their websites to include adequate information and advice about lifestyle for cancer survivors, or they risk cancer survivors turning to less reliable sources of information. Health professionals should be appropriately trained to deal with questions about lifestyle and to advise cancer survivors about lifestyle changes following their diagnosis.

## Introduction

### Background

There are more than 2 million people in the United Kingdom living with a cancer diagnosis, and this number is predicted to rise to over 5 million by 2040 [1]. Compared with the general population, cancer survivors are at a raised risk of cardiovascular disease, diabetes, osteoporosis, and second primary cancers [2,3]. Given these increasing numbers, addressing these long-term and late effects of cancer is an increasingly urgent issue to help relieve the burden on health services. In addition to being linked to cancer risk [4,5], smoking, poor diet, low levels of physical activity, and higher body weight have all been associated with increased risk of cancer recurrence and mortality in survivors [6-11], as well as influencing other major causes of morbidity and mortality. This has led to increasing interest in the role of lifestyle change as a means of improving long-term outcomes among cancer survivors.

There is a wealth of evidence linking lifestyle change, such as increasing physical activity levels, with improvements in quality of life and symptoms among cancer survivors [12]. Evidence is more limited for the impact on cancer outcomes, although there is emerging evidence that becoming physically active and intentionally losing weight (if overweight) may be associated with improvements in physiological markers among breast cancer survivors [12,13]. Also among breast cancer survivors, there is some evidence that following a low-fat diet post diagnosis may be associated with a reduced risk of cancer recurrence [14]. Further studies are underway to examine the impact of behavior change on survival [15], but nonetheless, findings to date highlight the potential of behavior change to improve long-term outcomes for cancer survivors.

### Prior Work

A cancer diagnosis has been considered as a candidate “teachable moment”: a time or setting at which motivation to adopt risk-reducing health behaviors is raised [16]. In line with this, surveys and qualitative studies have found that some cancer survivors report making positive lifestyle changes following their diagnosis, including eating more healthily [17-19] and being more physically active [19-21]. However, despite these reported changes, the health behaviors of cancer survivors in the United Kingdom have been found to be suboptimal, with only around 51% engaging in moderate physical activity, 15% continuing to smoke, and 8% consuming two or more alcoholic drinks per day [22]. Furthermore, studies that compare lifestyle change over time in groups that either do or do not receive a cancer diagnosis have failed to show evidence of sustained positive lifestyle changes following a cancer diagnosis and typically show a reduction in physical activity [23-25].

A range of factors may influence whether cancer survivors make lifestyle changes following their diagnosis. Mobility impairments, ill health, weather, and time have all been cited as barriers to exercise participation in this population [26,27], and unreliable information has been reported as a barrier to making dietary changes [28]. Lack of access to reliable information may partly be due to the absence of professional advice in the cancer context. This is consistent with surveys of health professionals that indicate that few of them discuss lifestyle factors such as physical activity with their cancer patients [29,30]. A recent survey of 3300 cancer survivors conducted by the UK Department of Health also found that over 20% of cancer survivors would like more advice on diet and lifestyle, suggesting that many of them are not receiving sufficient information on the topic [31]. This has been echoed by qualitative studies that have found cancer survivors report a lack of information about physical activity, diet, and weight [32].

If cancer survivors desire more information about lifestyle but do not receive much advice within the medical setting, they may choose to seek out information themselves, as was found in a qualitative study of colorectal cancer survivors in the United Kingdom [33]. Internet use is increasing among older adults. A recent report found that 53% of those over the age of 65 years are now online, and 70% of these use the Internet on a typical day [34]. Given that cancer is primarily a disease of older people [35], this age group comprises a large proportion of cancer survivors. A recent analysis of the Health Information National Trends Survey found that the Internet was the preferred source of information for 51% of cancer survivors, highlighting a shift from more traditional sources [36]. Similarly, breast cancer survivors have been shown to use the Internet for information, even after their treatment had ended; this was the most frequently cited source of information at 16 months post diagnosis [37]. This suggests that cancer survivors may desire and continue to search for information long after regular contact with their care team has ended. Another study found that cancer survivors were more likely to use the Internet to search for health-related purposes than the general population [38].

Qualitative research with breast and prostate cancer survivors in the United Kingdom suggests that those who use the Internet for information prefer non-commercial websites, and trust websites supported by the National Health Service (NHS) or other recognized “Centres of Excellence” such as charitable organizations and cancer centers [39]. Given the rising number of cancer survivors and the shift from health professional care to supported self-management [40], it is likely that such websites will increasingly be used to obtain information about a range of topics including lifestyle. Supporting self-management involves educating people about their condition and equipping them with the tools to help them choose healthy behaviors [41]. It is therefore crucial to examine the lifestyle information provided by these sectors in order to highlight any gaps and ensure that cancer survivors not only have access to reliable information but are provided with the tools to help them overcome barriers and make the behavior changes that could ultimately improve their long-term outcomes. If cancer survivors are unable to find the information they are looking for on these websites, they may turn to less reliable websites that put them at risk of misinformation.

### Aims of the Current Study

The purpose of this review was therefore to identify the lifestyle information and resources provided for cancer survivors by the statutory and charitable sectors and cancer centers in the United Kingdom. Specifically, we aimed to identify information on tobacco, physical activity, diet, weight, and alcohol designed for people who have been diagnosed with cancer. In addition to examining organizations that provide information to all groups of cancer survivors, this search also focused particularly on information specifically for patients diagnosed with breast, prostate, or colorectal cancer, as recent figures indicate that these constitute approximately 41% of new cancer diagnoses each year in the United Kingdom [42].

## Methods

### Identification of Statutory Sector Organizations

We first sought to identify any lifestyle information for cancer survivors provided by the UK Department of Health or NHS. The focus of this search was centered on the NHS Choices website [43], a Department of Health funded website that aims to provide objective and trustworthy information and guidance to the public on all aspects of health and health care. It is the largest health website in the United Kingdom and is certified by the Information Standard as a producer of reliable health and social care information [44].

### Identification of Cancer Centers

Comprehensive cancer centers accredited by the Organization of European Cancer Institutes (OECI) were also included in the search. This included cancer centers based in the NHS or in universities. As only a limited number of cancer centers are accredited by the OECI, this search was supplemented with a Google search for “cancer centre”, with cancer centers based in the NHS, charitable sector, or universities from the first page of results being included. Cancer centers in the private sector were excluded.

### Identification of Charitable Sector Organizations

The Charity Commission is the official register of charitable organizations in England and Wales [45]. Searches for generic, breast, prostate, and colorectal cancer charitable organizations were done separately using the advanced search function. To identify generic cancer charitable organizations the keyword “cancer” was searched for in “charity name”, “charity objects”, and “charity activities”. The search was refined by selecting only charitable organizations operating throughout England and Wales and those who described their operations as providing “advocacy/advice/information”. This was to ensure that the included voluntary sector organizations could reasonably be expected to provide advice on lifestyle. The three largest generic cancer organizations were selected from this list, provided they met the inclusion criteria outlined below. Organization size was defined by income in 2012; which was the information available from the Charity Commission. The three largest organizations were chosen as the researchers agreed these were the most publically well known in England and also appeared at the top of Internet search results. The search was then carried out using the keywords “breast cancer”, “prostate cancer”, and “colorectal cancer”, and the three largest organizations for each of these cancers was selected. The colorectal cancer search was repeated using the terms “bowel cancer”, “colon cancer”, and “rectal cancer”.

### Charitable Organization Inclusion Criteria

The inclusion criteria for charitable organization consisted of the following: registered in the Charity Commission database; within the top three breast, prostate, colorectal or generic (all cancer types) cancer charitable organizations in England (defined by income in 2012); listed in the Charity Commission database as providing advocacy, advice, or information; operating in England or Wales (there was no single category for England); aimed at adults; and colorectal and generic cancer charitable organizations must be for both men and women.

### Search for Lifestyle Information

The NHS Choices website was searched using the terms “cancer survivor”, “cancer AND physical activity”, “cancer AND exercise”, “cancer AND diet”, “cancer AND weight”, “cancer AND alcohol”, and “cancer AND smoking” in the website’s search function and manually searching the results and following relevant links. The same search was repeated in the websites of the cancer centers and charitable organizations but without the word “cancer”, as these sites were already specific to cancer information. If filters were available they were used to refine the results to pages aimed at cancer patients or survivors. If the website did not have a search function, a manual search of the site was conducted using the drop-down menus. The searches were conducted between November 2014 and January 2015.

### Lifestyle Information Inclusion Criteria

Information was included on physical activity, diet, weight management, alcohol, or smoking, aimed at improving the general or long-term health of cancer survivors. Lifestyle information designed to improve acute outcomes of cancer and its treatment (eg, manage a short-term diet problem or acute symptom management) was excluded as the focus was on longer-term survivorship. Information on cancer prevention was also excluded unless cancer survivors were specifically directed toward it.

### Data Synthesis

The initial searching of the 20 websites to identify lifestyle information for cancer survivors was conducted by KW, then a selection (N=4) was checked by FC. Any uncertainties or discrepancies were discussed and resolved with the other authors (RJB and AF). Once all the relevant lifestyle information had been agreed on, KW extracted the content. This included identifying any specific recommendations made by the organization and the basis of these recommendations. Other details about the information were also recorded including the format (eg, print, video, podcasts) and resources or advice for helping patients change their lifestyle behaviors.

## Results

### Statutory Sector Organizations

As outlined in the method section, the NHS Choices website was used to identify lifestyle information for cancer survivors provided by the UK government [43].

### Cancer Centers

Three comprehensive cancer centers in England were accredited by the OECI. These were the King’s Health Partners Integrated Cancer Center [46], the Cancer Research UK Cambridge Institute [47], and the Christie NHS Foundation Trust [48]. The top Google search results for “cancer centres” also found Maggie’s [49], University College Hospital Macmillan Cancer Centre [50], The Royal Marsden Hospital [51], and The Clatterbridge Cancer Centre [52].

### Charitable Sector Organizations

The search for generic cancer charitable organizations found 183 results. Once these had been narrowed down using the inclusion and exclusion criteria, the three largest charitable organizations were Cancer Research UK [53], Macmillan Cancer Support [54], and the World Cancer Research Fund (WCRF) [55]. The search for breast cancer, prostate cancer, and colorectal cancer charitable organizations found 13, 15, and 5 results respectively. The three largest for each cancer site were Breakthrough Breast Cancer [56], Breast Cancer Care [57], Breast Cancer Campaign [58], Prostate Cancer UK [59], Movember Europe [60], the Orchid Cancer Appeal [61], Bowel Cancer UK [62], Beating Bowel Cancer [63], and Bowel Cancer Information [64].

### Availability of Lifestyle Information

All the website searches yielded a large number of results, but the majority were not relevant. The NHS Choices website did not contain any lifestyle information for cancer survivors, but it did provide a link to a Cancer Research UK page on diet. It also included a page on lifestyle changes after chronic illness; however, this was not included as it did not specifically mention cancer. Ten organizations (3/7 cancer centers and 7/12 charitable organizations) had lifestyle information for cancer survivors available on their websites. Of these, the Christie NHS Foundation Trust [48], Macmillan Cancer Support [54], and Prostate Cancer UK [59] had the most comprehensive guides, covering physical activity, diet, weight management, smoking, and alcohol. Multimedia Appendix 1 shows a summary of the online lifestyle information provided by the different sources.

### Summary of Lifestyle Information

All ten organizations with lifestyle information for cancer survivors had information on diet and physical activity, but only seven had information on alcohol [48,51,54,55,57,59,63], six on weight management [48,51,54,55,57,59], and four on smoking [48,54,59,63] (Multimedia Appendix 1). The information from six organizations made reference to other guidelines; most often those produced by the WCRF [48,51,53-55,63]. Eight suggested discussing lifestyle with a health professional (including the general practitioner, cancer doctor, cancer nurse specialist, physiotherapist, or dietitian) before making any changes [48,51,53,54,57,59,62,63].

### Physical Activity

Ten organizations had information on physical activity. Eight of these provided specific recommendations on the duration and intensity of physical activity that cancer survivors should aim for [48,51,53-55,57,59,63], of which five recommended 150 minutes of moderate physical activity per week, in varying forms (eg, 30 minutes, 5 times per week) [51,53,54,57,59] and the other three recommended 30 minutes every day [48,55,63]. Some also highlighted the importance of reducing sedentary behavior [63]. Bowel Cancer UK and Maggie’s did not specify duration or intensity but emphasized the importance of being active [49,62].

Information about physical activity was provided in a variety of formats. Two had DVDs [54,57], one had a podcast [48], and others had booklets, leaflets, or factsheets available to download or order in paper formats [48,54,62,63]. Others had brief advice about becoming active on their own [51,53,59]. Some organizations offered exercise classes that patients could join to help them get active.

The majority of organizations gave suggestions on the types of physical activity cancer survivors could do, for example, walking, swimming, or housework [48,51,54,55,57,59,62], and some provided specific exercises for cancer survivors to try at home [48]. These often included information about the benefits of being physically active following a cancer diagnosis, for example, “exercise for cancer patients can reduce the risk of cancer coming back” [48,53,54,57]. Patients were encouraged to start exercise gently and build up slowly and some organizations gave examples of how to do this, for example, “5 minutes of housework in the morning followed by a 5 minute walk to the shop, followed by a 10 minute dog walk” [48]. Several organizations provided information about safety during exercise and when to be careful, for example, “people with low immunity should avoid public gyms” or “stop exercising if you feel sick or are sick during exercise” [48,53,54,62].

A range of resources were provided to help cancer survivors be physically active. The leaflets included case studies of patients with tips on exercising with cancer, and advice on finding local exercise programs. The DVDs had information on how to become more active, including advice from experts, case studies from other cancer survivors, and exercise demonstrations [54,57].

### Diet

All organizations recommended that cancer survivors eat a balanced diet, and the majority provided further details. They highlighted the importance of eating plenty of fruit, vegetables, and starchy foods, and limiting intake of energy dense foods (high in sugar or saturated fat) and red or processed meat. Prostate Cancer UK also provided a list of more specific and unusual foods that may be beneficial (eg, green tea and tomatoes), although they acknowledged that evidence is limited.

The websites provided information about diet in a range of formats. Five organizations had leaflets available for patients to download and print at home [48,51,59,62,63]. Others had videos for patients to watch [48,57] and podcasts for them to listen to [48]. In some cases, the website itself did not provide much information but had details about free courses patients could sign up to in order to learn more about diet [49].

All of the organizations with information about diet gave guidelines for what cancer survivors should be eating. Most provided a diagram of the “Eatwell plate” [65] to help cancer survivors understand the different food groups on which they should be basing their diet [48,51,57,59,62,63]. Some then gave examples of the types of foods that come under each food group, for example, “meat, fish, eggs, tofu, soya products, pulses and Quorn are a good source of protein” [48,51,62] and reasons why these foods are beneficial or harmful, for example, “fibre keeps bowels working regularly” or “red and processed meat are associated with an increased risk of some cancers”. In order to help cancer survivors eat appropriate amounts of different types of foods, several organizations gave examples of portion sizes, for example, a serving would be “three heaped tablespoons of cooked vegetables” [48,54,55,57]. To inspire patients, many organizations also provided recipe ideas for meals and snacks, for example, breakfast could be “wholegrain cereal topped with sliced banana and semi-skimmed milk” [48,51,54,55].

As well as this fairly general information on what to eat, several organizations provided information about what to eat following specific cancers or treatments, or when experiencing particular symptoms. For example, Beating Bowel Cancer provided an explanation of how bowel cancer treatment and surgery affects the bowel and how this may impact on diet [63]. They also included tips for eating and avoiding bowel symptoms, for example, “eat at regular intervals, and don’t eat on the move”. Other organizations gave information on what to eat when losing or gaining weight. For example, the Royal Marsden suggested that when losing weight, it is best to “eat when your appetite is best and have small regular meals” [51].

Some organizations provided some tools to help cancer survivors with their diet. For example, the Royal Marsden gave some tips for overcoming problems with eating, such as “if you are too tired, get friends to help with shopping or have snacks that don’t require much preparation” [51]. The Beating Bowel Cancer leaflet contained quotes from other patients with tips on what they found useful, for example, “Ginger beer really helped with nausea when undergoing chemotherapy”, as well as tips for family members [63]. Similarly, the Breast Cancer Care DVD was largely narrated by patients who told their stories about how they changed their diet following their cancer diagnosis [57].

### Weight Management

Seven organizations provided information on weight management for cancer survivors [48,51,54,55,57,59,62]. They all recommended maintaining a healthy weight (within the normal Body Mass Index range), and the WCRF recommended being as lean as possible without becoming underweight. Several organizations recommended that overweight people should try to lose their excess weight but emphasized that this should be done gradually (at around 0.5-1 kg a week) and should be done in consultation with a health professional [54,57,59,62]. In contrast, the Royal Marsden recommended that those who are overweight should not try to lose weight during treatment as this would make them more susceptible to infections and poor wound healing [51].

Several organizations provided advice on how to lose weight with a focus on healthy eating and physical activity. Four had advice on their websites to help people get started, including tips on weight loss (and weight gain for those who had lost weight during treatment) [51,54,57,59]. Two included information about weight in their booklets about diet [48,51].

### Alcohol

Seven organizations provided information on alcohol [48,51,54,55,57,59,63]. These were almost identical and recommended 2-3 units per day for women and 3-4 units for men (three organizations stated this as the number of drinks: 1 for women and 2 for men) [51,55,63]. The Christie NHS Foundation Trust did not provide a specific recommendation but recommended drinking less alcohol [48]. They did not provide much advice on how to limit alcohol consumption, but one (Prostate Cancer UK, 2014) referred to the NHS Choices website.

### Smoking

Four organizations provided information on smoking [48,54,59,63], recommending that smokers should quit. These organizations did not provide their own advice on how to stop smoking but referred smokers to smoking cessation services and the NHS Choices website for further support.

## Discussion

### Principal Results

The purpose of this review was to identify lifestyle information specifically for cancer survivors provided by the statutory and charity sectors in the United Kingdom. Ten organizations had lifestyle information for cancer survivors on their websites. The Christie NHS Foundation Trust [48], Macmillan Cancer Support [54], and Prostate Cancer UK [59] had the most comprehensive guides, covering physical activity, diet, weight management, smoking, and alcohol. The NHS website did not provide any lifestyle information for cancer survivors but had a link to Cancer Research UK’s information about diet.

The absence of lifestyle information for cancer survivors on the NHS website is a matter of concern, given that the NHS is the preferred source of information for many patients [39]. It is encouraging that the NHS Choices website provides links to Cancer Research UK’s webpage on diet, but it would be helpful if they also directed cancer survivors to advice on physical activity and other health behaviors. Although there was no information on the main NHS website, the Christie NHS Foundation Trust [48] had very comprehensive information on its website, suggesting that lifestyle information from statutory organizations is provided to cancer patients at a local level. However, not all cancer centers provided lifestyle information, which may lead to a geographical disparity in access to lifestyle information. Even if some cancer centers have lifestyle information on their websites, patients from other centers may not know it exists or where to find it.

In the charitable sector, Macmillan Cancer Support [54] and Prostate Cancer UK [59] had the most comprehensive information on their websites, consistent with their being leading cancer charities. Macmillan Cancer Support in particular had dedicated sections on its website, making it easy for cancer survivors to navigate and find the lifestyle information they need. Several of the other charitable organizations (eg, Cancer Research UK [53]) and Breast Cancer Care [57]) had information on each health behavior in a different section, making it more difficult to assemble the relevant information. This highlights a challenge that cancer survivors may face when searching for information about lifestyle.

### Comparison With Prior Work

Where lifestyle recommendations were given, they were similar to UK government guidelines for the general population [66-70]. They included not smoking, limiting alcohol intake, maintaining a healthy weight, being moderately physically active for at least 150 minutes per week, and eating a diet high in fruit and vegetables and low in fat, sugar, and red and processed meat. This is likely to be due to the lack of research evidence to inform development of specific recommendations for cancer survivors. In 2007, the WCRF reviewed the evidence for the role of diet and physical activity in both cancer prevention and survival and concluded that cancer survivors should follow general population recommendations for cancer prevention [5]. More recent study results have been generally consistent with these recommendations, demonstrating associations between cancer survival and physical activity [7,12], low-fat diet [14], not smoking [71], and limited alcohol consumption [72]. However, five organizations suggested that cancer survivors who are overweight or obese should attempt to lose weight [40,48,55,57,59]. This recommendation is less well supported by the literature as weight loss has been associated with poorer disease outcomes for cancer survivors, even among those who are overweight or obese [73,74]. In the absence of good trial evidence, organizations may choose to be cautious about recommending weight loss for cancer survivors and instead emphasize the importance of a healthy diet and physical activity. If they want to provide weight recommendations, the evidence suggests that avoiding weight gain would be preferable. The Royal Marsden’s recommendation was more in line with the evidence saying that it is not a good idea to lose weight during treatment, even if overweight [51]. Such inconsistencies in recommendations may be confusing for cancer survivors, particularly those who lose or gain weight during treatment.

### Implications

Our findings have considerable implications for the organizations included in this review. On the whole, the level of information provided was suboptimal, as only half of the organizations provided any information about lifestyle and only three provided information on all health behaviors. This was the case even though we included the NHS website and those of charitable organizations that all described their operations as providing “advocacy, advice or information”. These findings are concerning given that statutory and charitable sector organizations and cancer centers have been found to be a favored source of information for cancer survivors and are likely to be the first point of call for those seeking information [39]. With the rise of Internet use among older adults [34] and the increasing focus on supported self-management, the websites of these organizations are likely to experience increasing traffic [40]. However, if cancer survivors are unable to find the information they are looking for on these websites, they may turn to less reliable sources. Given the abundance of misreporting about lifestyle and cancer in the media and online [75], this would put cancer survivors at risk of misinformation and potentially hinder their chances of giving themselves the best long-term outcomes. As a result, it is crucial that the information on the majority of these websites is improved. Specifically, the main NHS website would benefit from including information about lifestyle specifically for cancer survivors, or alternatively add clear links to hospitals already providing good quality information and advice such as the Christie NHS Foundation Trust or the Royal Marsden. Other organizations would benefit from reorganizing their websites so that recommendations are easy to identify and all lifestyle information can be found in one section rather than having to search for behaviors separately [53,57].

Several of the organizations referred patients to other sources of information and emphasized the importance of talking to a health professional before making any lifestyle changes. The latter may be problematic for longer-term survivors as they may no longer have regular contact with their health care team. If patients are required to make an appointment with their general practitioner before making lifestyle changes, they may be less likely to make those changes, whether through loss of motivation or other barriers. Those who do have contact with their health care team may find that their doctor or nurse may be unable to advise them about lifestyle. Health professionals have reported lack of specialist knowledge about risk factors for cancer as a barrier to discussing lifestyle [76]. This made them reluctant to raise the issue of lifestyle change without the appropriate support to help patients make changes. It is therefore important to ensure that clinicians receive appropriate education about the importance of a healthy lifestyle following a cancer diagnosis and that they are trained in how to discuss these issues with patients. Provision should also be made for cancer survivors to speak to a specialist if they would like, such as a dietitian or exercise physiologist, where such referral schemes are in place. Another important source of information for cancer survivors is other survivors who may have already experienced particular issues with lifestyle. Some of the organizations had incorporated the experience of long-term cancer survivors into the information they provided in order to give a unique perspective and help motivate newer survivors.

This study also has implications for Internet research in general. Evaluating the content of these websites is crucial in order to hold organizations accountable for the information they provide. This may drive up quality in a way that anonymous feedback on a website may not. Evaluations such as these can drive positive change in Internet material and should be repeated at regular intervals to ensure that the quality of these websites continues to improve.

### Limitations

This study has a number of limitations. Although it included 20 UK-based statutory and charitable organizations and cancer centers, it is likely that users in the United Kingdom who search for information about lifestyle would also encounter websites based in North America or other English-speaking nations. Therefore, a wider search, incorporating all English-language websites, could be useful. Also, in addition to reviewing information by statutory and charity organizations, it may be useful to expand the evaluation to incorporate commercial organizations (eg, private health care companies) as users will potentially encounter them when searching for lifestyle information on the Internet. However, research has shown that patients do not favor such sites [39]. All searches were conducted between November 2014 and January 2015, and the organizations may continually update the information on their website. However, this review provides an important snapshot of the availability of lifestyle information for cancer survivors at present. Historically, charitable organizations may have websites in order to fundraise rather than provide information, which may explain the limited information available. However, all of the included charitable organizations described their operations as providing “advocacy/advice/information”, so they could reasonably be expected to provide such information. This review focused on the availability of lifestyle information online, but there are a range of other areas of information that are also important to cancer survivors such as psychological, sexual, and work-related issues, that were not included in this review.

### Conclusions

Although several organizations had some information on lifestyle for cancer survivors, there was no advice on the NHS website and only three organizations had comprehensive guides, encompassing diet, physical activity, weight, alcohol, and smoking. These organizations should consider adding or updating their websites to include adequate information and advice about lifestyle for cancer survivors, or they risk cancer survivors turning to less reliable sources of information. The majority of recommendations emphasized that cancer survivors should talk to a health professional before making any lifestyle changes. Health professionals should be appropriately trained to deal with questions about lifestyle and to advise cancer survivors about lifestyle changes following their diagnosis.

## Appendix

Multimedia Appendix 1 Summary of online lifestyle information for cancer survivors.

## Abbreviations

NHS: National Health Service
OECI: Organization of European Cancer Institutes
WCRF: World Cancer Research Fund
