# Supplementary material for: Availability of Information About Lifestyle for Cancer Survivors in England: A Review of Statutory and Charitable Sector Organizations and Cancer Centers
Source: JMIR Cancer. 2015 Mar 9;1(1):e2. doi: 10.2196/cancer.3521 (PMC5367671; doi:10.2196/cancer.3521)
Supplement: Multimedia Appendix 1 [file cancer_v1i1e2_app1.pdf]

Multimedia Appendix 1: Summary of online lifestyle information for cancer survivors

Organization	Physical activity	Diet	Weight management	Alcohol	Smoking	Which guidelines is the information based on?	Which sources does the information direct to?
NHS http://www.nhs.uk/Pages/HomePage.aspx	None	None	None	None	None	N/A	Provides link to CR-UK page on diet
King's Health Partners Integrated Cancer Centre http://www.kcl.ac.uk/lsm/icc/index.aspx	None	None	None	None	None	N/A	N/A
Cancer Research UK Cambridge Institute http://www.cambridgecancer.org.uk/	None	None	None	None	None	N/A	N/A
The Christie NHS Foundation Trust http://www.christie.nhs.uk/	Recommendation:<br>- Exercise and stay active. Try to accumulate at least 30 minutes of physical activity throughout the day. During the 30 minutes you	Recommendation:<br>- Eat a balanced diet. Reduce your intake of high energy foods and sugary drinks, eat 5 portions of different coloured fruit	Recommendation:<br>- The aim following treatment is to be as near to a normal healthy weight as possible. If you have experienced	Recommendation:<br>- Drink less alcohol. Drinking less alcohol can help improve mood and sleep, and reduce nausea and a sore mouth during	Recommendation:<br>- Go smoke free. Becoming smoke-free is the most important step you can take to reduce ill health and early death from many	Department of Health alcohol guidelines	Doctor, physiotherapist, dietitian, nurse, Macmillan, WCRF, Netfit, CR-UK, National Association of Cancer Exercise Rehabilitation, NHS Choices, Drink Aware, British

Organization	Physical activity	Diet	Weight management	Alcohol	Smoking	Which guidelines is the information based on?	Which sources does the information direct to?
	must work at moderate intensity, meaning you are very slightly breathless.<br>- Exercise is not contraindicated for people living with or recovering from cancer. Research suggests daily exercise can help with reducing nausea, help with fatigue, insomnia and low mood. It can boost confidence and self-esteem, reduce anxiety and depression, help with lymphedema and ease pain.<br>- Can download 'Be Active, Stay Active' booklet which contains information on	and vegetables a day, eat less red and processed meats, eat less salty foods and processed foods and increase your fibre foods.<br>- Having a healthy eating approach to your diet can reduce the risk of cancer reoccurring or development of a new one and reduce risk of other diseases such as heart disease.<br>- Can download 'Eating well following treatment and recovery from	weight gain, aim to reduce it gradually by following a healthy eating plan.<br>- Can download 'Eating well following treatment and recovery from cancer' booklet which contains information (not for people who are underweight) on foods associated with weight gain/loss.<br>- Can download 'Be Active, Stay Active' booklet which contains information on	chemotherapy and radiotherapy.<br>- Includes information on why hospital staff will assess alcohol levels and some common facts and myths about alcohol.<br>- Can download 'Smoking Cessation and Alcohol Advice Services' leaflet	cancers and other lung and heart diseases. We strongly advise you not to smoke.<br>- Research has shown across a range of cancers that Going Smoke Free will help improve fatigue levels, sleep, pain, nausea, help with treatment recovery and reduce the risk of developing new cancers.<br>- Can download 'Smoking Cessation and Alcohol Advice Services' leaflet		Dietetic Association, Manchester Community Alcohol Team, 'Quit' – smokers quitline

Organization	Physical activity	Diet	Weight management	Alcohol	Smoking	Which guidelines is the information based on?	Which sources does the information direct to?
	the importance of exercise, how much to do, examples of exercises, as well as directing to further sources of information and support.<br>- Has a podcast on exercise.	cancer' booklet which contains information on what foods to eat and why, sample menus and tips, and directs patients to further sources of information.<br>- Has a podcast called 'Let's move forward with your eating' by a specialist dietitian.	the risks of being overweight and how exercise can help maintain weight.<br>- Has a podcast called 'Worried about losing weight' by a specialist dietitian.	which contains information and directs patients to alcohol advice and support services.	which contains information on the harms of smoking, advice on e-cigarettes and directs to smoking cessation and support services.		
Maggie's https://www.maggiescentres.org/	- No specific recommendation but says that evidence shows that exercise offers many benefits, from reducing fatigue and improving your wellness and physical fitness, to building your confidence during and after	- No specific recommendation but says that eating well during and after cancer treatment can make a real difference to the way you feel.	None	None	None	N/A	Maggie's exercise classes and nutrition workshops, Maggie's Online Centre

Organization	Physical activity	Diet	Weight management	Alcohol	Smoking	Which guidelines is the information based on?	Which sources does the information direct to?
	cancer treatment.<br>- Maggie's offers exercise classes for people with cancer so there is information on how to book these on their website.	- Maggie's offers nutrition workshops for people with cancer so there is information on how to book these on their website.					
University College Hospital Macmillan Cancer Centre https://www.uclh.nhs.uk/OurServices/OurHospitals/UCH/CC/Pages/Home.aspx	None	None	None	None	None	N/A	N/A
The Royal Marsden Hospital http://www.royalmarsden.nhs.uk/pages/home.aspx	Recommendation:<br>- 30 minutes of physical activity five times a week.	Recommendation:<br>- Reduce your intake of high-calorie foods and avoid sugary drinks, eat at least five portions of	Recommendation:<br>- Maintain your weight within the normal BMI range. If overweight, it is not good to lose weight during	Recommendation:<br>- Limit your alcohol intake to two drinks a day for men or one drink a day for women.	None	Department of Health exercise and diet guidelines, WCRF diet guidelines	Doctor, nurse, physiotherapist, dietitian, Macmillan Cancer Support, Department of Health, Cancer Equality

Organization	Physical activity	Diet	Weight management	Alcohol	Smoking	Which guidelines is the information based on?	Which sources does the information direct to?
		fruit/vegetables every day, eat a portion of pulses or wholegrain foods with every meal, reduce your intake of red meat to no more than 500g (18oz) a week and eat minimal amounts of processed meats, lower your salt intake, do not use dietary supplements for the prevention of cancer.	treatment as it may make you more susceptible to infections and poor wound healing.				
	- Provides examples of different types of activities (e.g. walking, gardening) and emphasises starting slowly and building up gradually.	- Provides examples of different foods to eat and foods to avoid.	- Provides information on how to calculate body mass index (BMI)	- Provides information about the number of units in different alcoholic drinks.			
		- Can download ‘Eating well when you have	- Can download ‘Eating well when you have				

Organization	Physical activity	Diet	Weight management	Alcohol	Smoking	Which guidelines is the information based on?	Which sources does the information direct to?
		cancer' booklet which contains information on different foods, meal ideas, recipes, advice on overcoming problems with eating, frequently asked questions and directs patients to other sources of information.	cancer' booklet which includes advice on what to eat if you are losing/have lost weight and what to do if you are overweight.				
The Clatterbridge Cancer Centre http://www.clatterbridgecc.nhs.uk/	None	None	None	None	None	N/A	N/A
CR-UK http://www.cancerresearchuk.org/	Recommendation:<br>- No general UK guidelines about exercising after cancer but generally doctors advise 30 minutes a day, 5 days a week.	Recommendation:<br>- Important to include everything you need in your diet including protein, carbohydrates, fat, vitamins and minerals, water and fibre.	None	None	None	American College of Sports Medicine (ACSM)	Macmillan Cancer Support, Cancer doctor/nurse

Organization	Physical activity	Diet	Weight management	Alcohol	Smoking	Which guidelines is the information based on?	Which sources does the information direct to?
	- Exercise should be tailored to the individual patient.<br>- Build up level of exercise gradually and don't do too much in one day. Gentle walking or swimming is fine for just about everyone.	- No advice on how to eat a healthy diet or make dietary changes for long-term health.					
Macmillan Cancer Support http://www.macmillan.org.uk/Home.aspx	Recommendation:<br>- UK adults advised to do 2.5 hours moderate intensity activity per week.<br>- A 'Move More'	Recommendation:<br>- There still isn't enough clear information to make exact recommendations about what someone with cancer should eat. In general, cancer experts recommend following a healthy, balanced diet.<br>- A balanced diet	Recommendation:<br>- Try to keep your weight within the normal range.<br>- Aim for a	Recommendation:<br>- Limit alcohol intake and include one or two alcohol-free days per week.<br>- Men should	Recommendation:<br>- If you're a smoker, choosing to stop is a decision that will benefit your health.<br>- Refers to	ACSM, WCRF, British Heart Foundation, Drinkaware, National Comprehensive Cancer Network, NHS Choices, The Organic Center, ACSM, National Cancer Survivorship Initiative (NCSI), Department of Health: healthy lives healthy people, Food Standards Agency, NICE, academic papers	GP, nurse specialist, NHS Smokefree/ smoking cessation services, Department of Health, World Health Organization

Organization	Physical activity	Diet	Weight management	Alcohol	Smoking	Which guidelines is the information based on?	Which sources does the information direct to?
	pack is available to order and includes a booklet with tips for becoming active, case studies and an activity planner (goal setting and diary).<br>- Also have a DVD called 'Get Active, Feel Good' which includes exercise demonstrations and case studies.	includes lots of fruit and vegetables, plenty of starchy foods, some protein-rich foods, some milk and dairy foods, small amounts of foods high in fat, salt and sugar, and sugar-free drinks.<br>- If you're thinking of taking dietary supplements, it's important to talk to your cancer doctor first<br>- Can download 'Health eating and cancer' leaflet which contains these recommendation s, advice on making changes, and frequently asked questions.	maximum weight loss of 0.5-1kg per week.<br>- Advises speaking to GP before trying to lose weight.<br>- Includes advice on health eating and activity and has a 'food and activity planner' available to download.	avoid drinking more than 3-4 and women 2-3 units per day.	smoking cessation services, NRT and medications for help with quitting.		
WCRF http://www.w	- Evidence is not clear enough to	- Evidence is not clear enough to	- Evidence is not clear enough to	- Evidence is not clear enough to	None	WCRF recommendations for	N/A

Organization	Physical activity	Diet	Weight management	Alcohol	Smoking	Which guidelines is the information based on?	Which sources does the information direct to?
crf-uk.org/	make detailed recommendations for cancer survivors. Until further research is done, the best advice to reduce the risk of cancer returning is to follow recommendations for prevention.<br><br>Recommendation for prevention:<br>- Move More. Aim for 30 minutes or more of moderate (or vigorous) activity a day. **Reduce the time you spend doing sedentary activities**	make detailed recommendations for cancer survivors. Until further research is done, the best advice to reduce the risk of cancer returning is to follow recommendations for prevention.<br><br>Recommendations for prevention:<br>1) Avoid high calorie foods and drinks, 2) Put plant foods first, 3) Eat less red meat and cut down on processed meats, 4) Eat less salt, 5) For most people, eating a healthy, balanced diet is a better way of reducing your cancer risk than taking supplements. If you need more advice about taking supplements, it is best to talk to your	make detailed recommendations for cancer survivors. Until further research is done, the best advice to reduce the risk of cancer returning is to follow recommendations for prevention.<br><br>Recommendation for prevention:<br>- Stay in shape. If you're overweight, losing even a few pounds will make a positive difference to your health**.**	make detailed recommendations for cancer survivors. Until further research is done, the best advice to reduce the risk of cancer returning is to follow recommendations for prevention.<br><br>Recommendation for prevention:<br>- Drink less alcohol. Aim for no more than two drinks a day if you're a man and no more than one drink a day if you're a woman.		cancer prevention, Department of Health	

Organization	Physical activity	Diet	Weight management	Alcohol	Smoking	Which guidelines is the information based on?	Which sources does the information direct to?
		GP					
Breakthrough Breast Cancer http://www.breakthrough.org.uk/	None	None	None	None	None	N/A	N/A
Breast Cancer Care http://www.breastcancercare.org.uk/	Recommendation:<br>- 150 minutes of moderate activity per week.<br>- Before you start exercise it's important to discuss it with your specialist team and build up gradually.<br>- Includes tips on how to get active	Recommendation:<br>- Try to enjoy a balanced, nutritious diet with plenty of fruit and vegetables, plenty of wholegrain starchy foods, some milk and dairy foods and some lean protein.<br>- Can order 'Eat well, keep active after breast cancer' DVD which includes case studies, tips on eating well and advice from experts.	Recommendation:<br>- If you decide to lose weight after treatment, aim to lose 0.5-1kg per week.<br>- Recommends talking to dietitian or GP.<br>- Includes tips for losing and	Recommendation:<br>- 2-3 units of alcohol a day or women and 3-4 for men.	None	N/A	Specialist team, GP, Dietitian, NHS Choices

Organization	Physical activity	Diet	Weight management	Alcohol	Smoking	Which guidelines is the information based on?	Which sources does the information direct to?
	including joining their walking groups 'Best Foot Forward'.<br>- Can order 'Eat well, keep active after breast cancer' DVD which includes a fitness class, advice from experts and case studies.		gaining weight depending on patient requirements.				
Breast Cancer Campaign http://www.breastcancercampaign.org/	None	None	None	None	None	N/A	N/A
Prostate Cancer UK http://prostatecanceruk.org	Recommendation:<br>- Aim to be physically active at least 2-3 times per week. Start gently and build up to 30 minutes of moderate exercise 3-5 days per week.	Recommendations:<br>- Eat at least 5 portions of fruit and vegetables a day, about 1/3 of diet should be starchy foods, include some protein, include some dairy foods, eat foods that are low in fat or saturated	Recommendation:<br>- If overweight, eating a balanced diet, cutting down on fatty and sugary foods and being active will help to lose weight gradually and healthily.	Recommendation:<br>- Men should not regularly drink more than three to four units of alcohol a day.	Recommendation:<br>- Stop smoking	None reported	Dietitian, NHS Choices, British Dietetic Association, British Nutrition Foundations

Organization	Physical activity	Diet	Weight management	Alcohol	Smoking	Which guidelines is the information based on?	Which sources does the information direct to?
		fat, cut down on foods high in sugar, cut down on salt, drink 6-8 glasses of water per day.					
	- Gives some simple exercise ideas but recommends speaking to GP or hospital doctor before starting an exercise plan.	- Foods that may be beneficial are: soy and pulses, green tea, tomatoes, selenium (not supplements), cruciferous vegetables, pomegranate juice, fish.	- Recommends asking GP to refer to a dietitian or weight loss programme.	- Suggests looking at NHS Choices website for advice on managing alcohol consumption.	- Stopping smoking can reduce the side effects of treatment.		
	- Can download a 'diet, physical activity and prostate cancer' factsheet which includes information on all 5 lifestyle factors.	- Foods to limit: dairy, red and processed meat, well done meat and fat.	- Can download a 'diet, physical activity and prostate cancer' factsheet which includes information on all 5 lifestyle factors.	- Can download a 'diet, physical activity and prostate cancer' factsheet which includes information on all 5 lifestyle factors.	- Suggests looking at NHS Choices website for advice on how to stop smoking.		
		- Gives some tips on healthy eating but recommends asking GP to refer to dietitian. Also suggests			- Can download a 'diet, physical activity and prostate cancer' factsheet which includes information on		

Organization	Physical activity	Diet	Weight management	Alcohol	Smoking	Which guidelines is the information based on?	Which sources does the information direct to?
		looking at NHS Choices website.<br>- Can download a 'diet, physical activity and prostate cancer' factsheet which includes information on all 5 lifestyle factors.			all 5 lifestyle factors.		
Movember Europe http://uk.movember.com/?home	None	None	None	None	None	N/A	N/A
Orchid Cancer Appeal http://www.orchid-cancer.org.uk/	None	None	None	None	None	N/A	N/A
Bowel Cancer UK http://www.bowelcanceruk.org.uk/	Recommendation:<br>- People living with and beyond cancer are now encouraged to remain active and resume daily activities as soon as possible	Recommendation:<br>- After bowel surgery, aim to eat a balanced diet.	- Does not include a specific recommendation but there are two leaflets available to download, one on 'gaining weight safely'	None	None	N/A	Macmillan Cancer Support, Penny Brohn Cancer Care, GP

Organization	Physical activity	Diet	Weight management	Alcohol	Smoking	Which guidelines is the information based on?	Which sources does the information direct to?
	during and after their treatment. Suggests building up to 30 minutes per day.<br>- There are two leaflets available to download including a 'staying healthy after bowel cancer' factsheet and a leaflet on 'physical activity after bowel cancer' which includes information on the benefits of physical activity, tips on building up and a case study.<br>- Recommends speaking to doctor or nurse before starting an exercise regime.	- There are several leaflets available to download including a 'staying healthy after bowel cancer' factsheet and a leaflet on 'what is a balanced diet', among other leaflets aimed at specific diet issues.<br>- These leaflets cover information the different food groups, advice on eating fibre, and information about vegetarian and vegan diets. They also have	and another on 'losing weight safely'.<br>- These leaflets have advice on how to gain and lose weight but do not state what constitutes a healthy weight or the amount of weight gain/loss to aim for.				

Organization	Physical activity	Diet	Weight management	Alcohol	Smoking	Which guidelines is the information based on?	Which sources does the information direct to?
		advice on what to eat at different stages of disease and treatment.<br>- Refers to Macmillan Cancer Support and other charitable organizations for more specific information.					
Beating Bowel Cancer https://www.beatingbowelcancer.org/	- Take 30 minutes of moderate exercise every day and avoid sitting for long periods.<br>- This information is included in the downloadable 'Living well after bowel cancer'	Recommendations: 1) Reduce the amount of processed foods, especially those high in fat and sugar, 2) Increase the amount of fresh fruit and vegetables, 3) Eat less than 500g red meat per week and avoid processed meat, 4) Eat small portions of high quality protein.<br>- This information is included in the downloadable 'Living well' and 'Eating Well'	N/A	Recommendation:<br>- If consumed at all, alcohol should be limited to 2 small drinks for men and 1 for women per day.<br>- This information is included in the downloadable 'Living well after bowel cancer'	Recommendation:<br>- Giving up smoking with have many health benefits, including reducing your risk of cancer.<br>- This information is included in the downloadable 'Living well after bowel cancer'	WCRF	GP, specialist nurse, NHS Choices, British Dietetic Association

Organization	Physical activity	Diet	Weight management	Alcohol	Smoking	Which guidelines is the information based on?	Which sources does the information direct to?
	leaflet.<br>- Suggests speaking to GP, specialist nurse or local sports center for information on local exercise classes.<br>- Also suggests a website with information on 'health walks' and the NHS Choices website.	leaflets which include tips from bowel cancer patients.		leaflet.	leaflet.		
Bowel Cancer Information http://www.bowelcancer.tv	None	None	None	None	None	N/A	N/A
